# PROGgeneV2: enhancements on the existing database

**DOI:** 10.1186/1471-2407-14-970

**Published:** 2014-12-17

**Authors:** Chirayu Pankaj Goswami, Harikrishna Nakshatri

**Affiliations:** Clinical Genomics, Thomas Jefferson University Hospitals, 1025 Walnut St, Suite 411, Philadelphia, PA 19128 USA; Department of Surgery, IU School of Medicine, 980 W. Walnut St., R3 C218E, Indianapolis, IN 46202 USA

**Keywords:** Biomarker, Multiple cancer, Survival, Pan cancer, Prognostic, mRNA, Database, Kaplan, Meier, KM

## Abstract

**Background:**

We recently published PROGgene, a tool that can be used to study prognostic implications of genes in various cancers. The first version of the tool had several areas for improvement. In this paper we present some major enhancements we have made on the existing tool in the new version, PROGgeneV2.

**Results:**

In PROGgeneV2, we have made several modifications to enhance survival analysis capability of the tool. First, we have increased the repository of public studies catalogued in our tool by almost two folds. We have also added additional functionalities to perform survival analysis in a variety of new ways. Survival analysis can now be performed on a) single genes b) multiple genes as a signature, c) ratio of expression of two genes, and d) curated/published gene signatures in new version. Users can now also adjust the survival analysis models for available covariates. Users can study prognostic implications of entire gene signatures in different cancer types, which are searchable by keywords. Also, unique to our tool, in the new version, users will be able to upload and use their own datasets to perform survival analysis on genes of interest.

**Conclusions:**

We believe, like its predecessor, PROGGeneV2 will continue to be useful for the scientific community for formulating research hypotheses and designing mechanistic studies. With added datasets PROGgeneV2 is the most comprehensive survival analysis tool available. PROGgeneV2 is available at http://www.compbio.iupui.edu/proggene.

**Electronic supplementary material:**

The online version of this article (doi:10.1186/1471-2407-14-970) contains supplementary material, which is available to authorized users.

## Background

Survival analysis using gene expression to derive predictive gene signatures is a commonly used feature in research studies employing high throughput genomic data. Gene signatures predictive of overall, relapse free or metastasis free survival are popular and several such signatures are published periodically and the data submitted to public repositories. Data from such studies which is available on the public domain can be leveraged to identify prognostic markers in different cancer types. We recently published ‘PROGgene’ [1], a tool that allows researchers to be able to use publicly available data to study prognostic implications of genes of interest in multiple cancers. This tool can be used to conduct on the fly survival analysis and create survival plots (Kaplan Meier, KM plots) based on gene expression of user input genes in user selected datasets from multiple cancers. Since its publication, PROGgene has received encouraging response. In the first version of the tool, we catalogued data from sixty-four studies from eighteen major cancer types. The data was primarily downloaded from Gene Expression Omnibus and The Cancer Genome Atlas. Version 1 of the tool had several areas for improvement. In this paper, we present the second release of PROGgene, PROGgeneV2, which has several enhancements over the previous version. PROGgeneV2 will be available freely for research and non-commercial use at the same URL as its predecessor, http://www.compbio.iupui.edu/proggene. In the new version, we have added additional datasets for existing and new cancer types. We have also added more functionality to the tool which will allow researchers to perform multifaceted survival analysis.

## Implementation

### New and additional datasets

**Figure 1:**
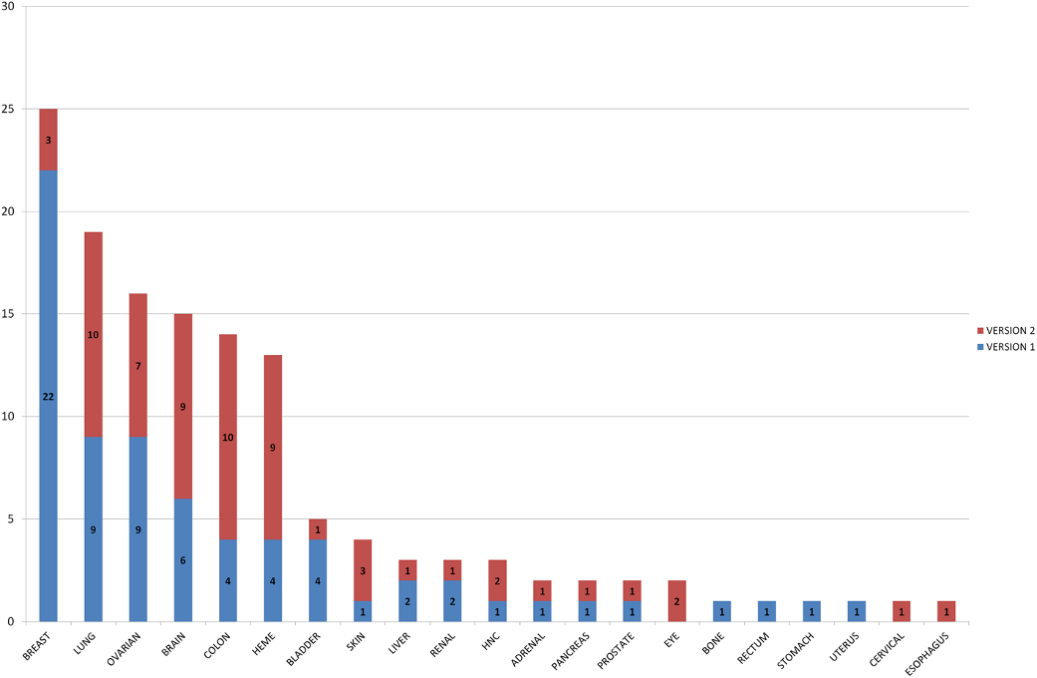
**Comparison of number of datasets available in PROGgeneV1 and V2.**

**Figure 2:**
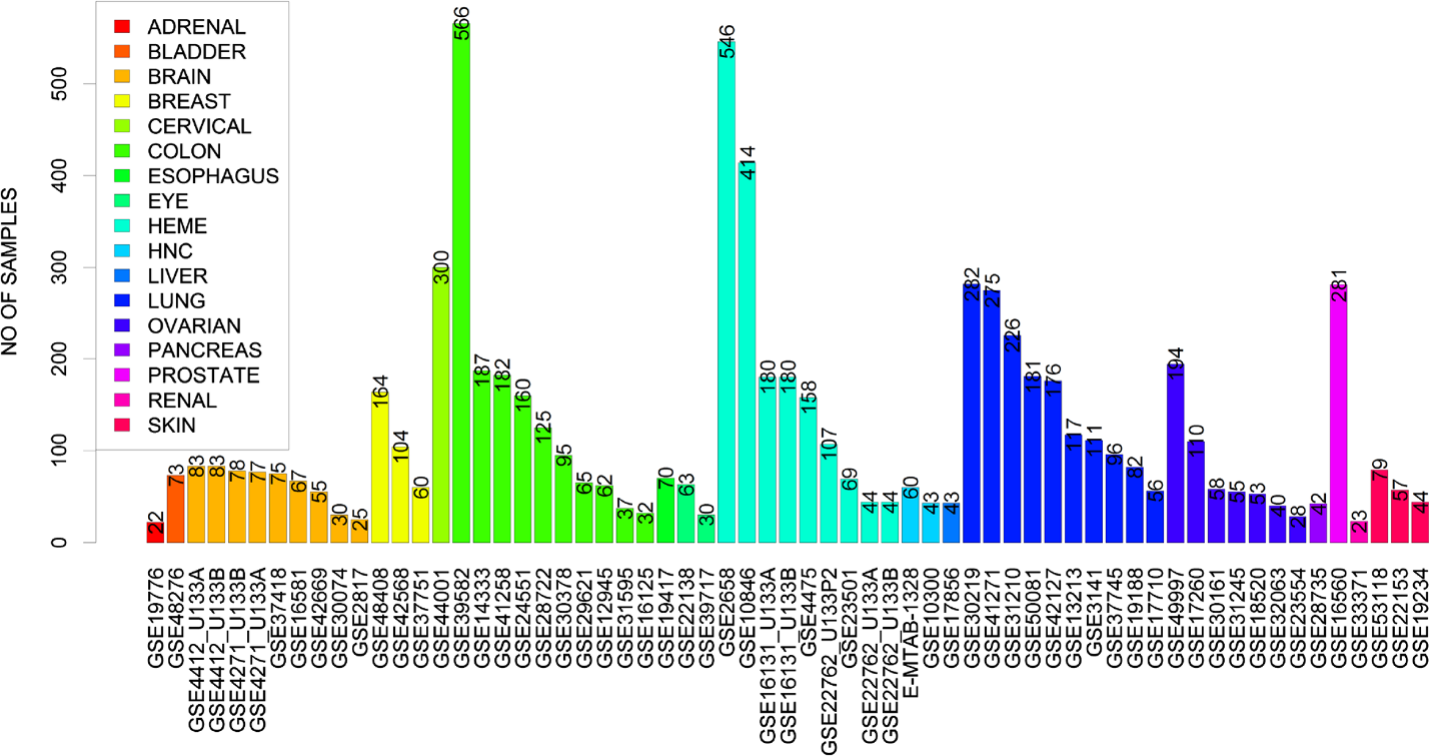
**Number of samples in datasets that are included in PROGgeneV2.**

The data in PROGgeneV2 are downloaded from published studies where data has been deposited to public repositories. The studies included have acquired proper authorization to release data based on human subjects. The first release of PROGgene catalogued sixty four cohorts from eighteen cancer types. In the new version, we have expanded the repository of the tool more than 2 folds by adding additional datasets. The tool now contains data from 134 cohorts from 21 cancer types. Figure 1 shows the number of datasets available in PROGgeneV1 and V2. Ten new datasets have been added for Lung and Colon cancers each in the new version. Datasets for Esophageal, Cervical and Eye cancers have been included for the first time. Figure 2 shows the number of samples in datasets that are included in the new version. Additional file 1: Table S1 lists the new datasets available in PROGgeneV2. For survival variables and covariates available for the new datasets, please refer to Additional file 1: Table S2.

### New features

In the first version of PROGgene, users were not able to study prognostic implications of ratio of expression of two genes. In the new version of PROGgene, we have added capability to study prognostic implications of a gene expression ratio. Users can input two genes in the home page and create survival plots for the gene expression ratio in cohorts of interest. In the new version, we have also added capability where users can create survival plots for published/curated gene signatures. We have included approximately ten thousand published/curated gene signatures from Molecular signature database [2] to our repository. These signatures include those from curated databases such as Gene Ontology [3], KEGG [4], Biocarta and Reactome [5] and from published studies. Users can do a keyword search on the gene signatures and the application retrieves genes included in the gene signature as provided by the Molecular Signature Database. When using gene signatures, only a combined plot using mean of expression of all genes in the signature is created for the entire signature.

Also added in the new version is the functionality to adjust survival models for covariates. Covariate data such as clinical variables is submitted along with gene expression data in public repositories. We have included the covariates available for datasets in the new version of the application. Survival models can be adjusted for multiple covariates. In PROGgeneV1, users were able to only divide the cohort into subgroups based on covariates and study survival implications of these genes in the subgroups, eg Stages I, II and III etc. This feature still remains in the new version.

Another important improvement made in the new version is capability to upload custom datasets. Users can now upload their own datasets to PROGGeneV2. The instructions to upload are provided on the application website. Upon upload of custom datasets, users can use custom datasets just like any default dataset on PROGGeneV2 to conduct survival analysis on genes in their dataset. Also, the prognostic plots in the new version are accompanied with summary statistics, such as no of events, median survival times for survival categories, confidence intervals etc. Lastly, in the new version we have also provided option for bifurcating the gene expression variable for proportional hazards analysis using mean, median, 25^th^ percentile or 75^th^ percentile of gene expression.

### Workflow

**Figure 3:**
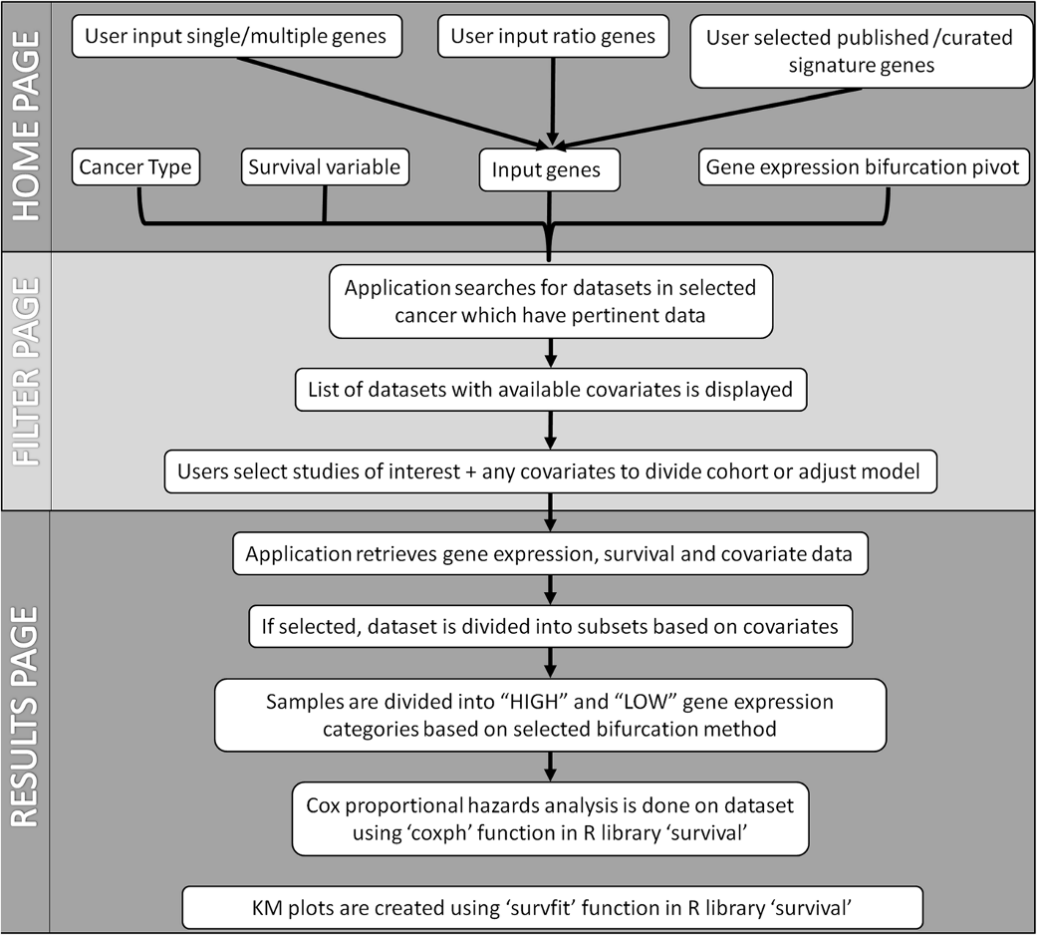
**A line diagram depicting workflow of PROGgeneV2 program.**

Similar to its predecessor, PROGgeneV2 is written in PHP5 with a MySQL database backend which stores gene expression data, covariates data and metadata for catalogued studies in form of relational database tables. Survival analysis is done using backend R script which employs R library ‘survival’ to perform Cox proportional hazards analysis (function ‘coxph’) and to plot prognostic plots (function ‘survfit’). The functioning of website is very similar to the previous version. General workflow of the application is presented in Figure 3. In first step users input the genes of interest on the ‘home page’. Users also select analysis method, e.g., single gene analysis, combined analysis for multiple user entered genes, analysis for gene-gene ratio, or combined analysis for genes from searchable published gene signatures at this step. In case of published gene signatures users can input keyword(s) in the search box which displays a list of published gene signatures having that keyword(s) along with number of genes per geneset. Users can then choose gene signature of their interest and list of genes for that signature is used in survival analysis as a combined gene signature. Also, at this step users select cancer type (in which they want to study prognostic implications of input genes), survival variable (death, metastasis, relapse) and gene expression bifurcation method (mean, median, 25^th^ percentile, 75^th^ percentile). Upon submission of this information, the application retrieves from the backend database list of all studies in selected cancer type which have a) gene expression data for input genes, b) survival data for selected survival variable and c) any covariates. This list is displayed on the ‘filter page’. In case of gene ratio analysis, only those studies are retrieved in which gene expression data is available for both entered genes. In case of single/multiple gene analysis or gene signature analysis, list of all studies available which have data for chosen survival variable is displayed irrespective of input genes, and genes from the input gene list that are present or absent in the study are displayed against each study. Users may accept or reject a study to be used in further analysis based on number of entered genes present/absent in that study. Also displayed on the filter page are covariates for each study. Users can choose covariates to divide the data into subsets or to adjust the survival model. Division of cohort and adjusting of data both are not allowed for same study. The application takes this information and retrieves study datasets from backend MySQL database. The datasets contain gene expression data, survival variable data and any covariate data for studies selected in the previous step. The datasets are then sent to the backend R script for survival analysis and creation of survival plots.

Based on input in home page and filter page the R script computes a final gene expression variable. Final gene expression could be a ratio of expression of two genes if the analysis method is gene ratio, or mean of gene expression of multiple genes if the analysis method is gene signature or if users input their own set of genes and wish to plot a combined prognostic plot for all genes instead of single gene, or gene expression of single gene if users selected to perform survival analysis on individual genes separately. The datasets also contain survival data in form of binary survival event variable and continuous time to event variable. Survival analysis is then performed on the final variables using cox proportional hazards analysis. For any study, if any covariates for dividing data selected in filter page, data at this step may be divided into subsets based on selected covariates, and survival analysis is performed on each subset separately. Conversely, users may select to adjust the survival model for available covariates for particular datasets, in which case selected covariates are added to the cox proportional hazards model with gene expression variable. For creation of Kaplan Meier (KM) plots, gene expression variable is bifurcated based on median, mean, 25^th^ percentile or 75^th^ percentile as selected by users in home page, and samples are categorized into having ‘HIGH’ or ‘LOW’ gene expression categories. KM plots are then created using ‘survfit’ function in library ‘survival’. Hazard ratio, log rank p value, lower and upper confidence intervals of hazard ratio retrieved from the result of proportional hazards analysis are displayed on the survival plot legend. The survival plots are stored on the server in png format and displayed on the results page. The results page is divided into separate sections for separate studies. Survival plots generated from each study's dataset are displayed in corresponding sections.

## Results and discussion

PROGgeneV2 has several advancements over its predecessor. One important feature is enabling study of prognostic implications of gene-gene expression ratio. Gene expression ratio's, for e.g., HOXB13 and IL17B ratio in early stage breast cancer has been identified as bearing prognostic importance [6]. With implementation of this feature, users will be able to study hypothetical gene ratios in multiple cancer types, and also published gene ratio biomarkers in other cancer types.

Researchers often focus on prognostic effects of entire gene signatures in cancers. For e.g., overexpressed WNT/CTNNB1 pathway is suggested to be prognostically unfavorable in terms of overall survival in high risk ovarian cancer patients [7]. Using PROGgene we validated unfavorable overall survival probability for samples with higher than median expression of WNT/CTNNB1 pathway genes (See Additional file 1: Figure S1). In the new version, users will be able to select gene signatures of interest from a list of approximately 10000 published or curated gene signatures compiled from the molecular signature database. Upon selection of a gene signature, average gene expression for all the genes in the signature is used to perform survival analysis in datasets.

In the new version we have also added the capability to upload custom datasets. Users can upload their own gene expression datasets to the tool and perform survival analysis on genes of interest. We have kept pre-formatting of datasets for upload very simple and instructions to do the same are provided on the application website. Users can upload a maximum of 3 custom datasets to the tool. Users will have to create an account on the website in order to upload custom datasets. This feature will enable users to analyse their own gene expression data to conduct survival analysis and comparing results with published studies.

## Conclusions

Several tools are available to conduct online survival analysis on genes of interest using publicly available data. But every tool lacks in some features, which renders its use limited for conducting in depth survival analysis. The caveats present in other tools motivated us to create PROGgeneV1 and now PROGgeneV2. PROGgeneV2 offers several advantages over contemporary survival analysis tools, such as ITTACA [8], Prognoscan [9] and KMPlot [10]. KMPlot is available primarily for 4 Cancer types only. The application also merges datasets exclusively generated on Affymetrix technology only to obtain larger sample cohort. A disadvantage of merging datasets from different studies is that it may cause over-fitting of data and erroneous results while studying biomarkers identified using other platforms such as Illumina technology [1]. ITTACA comprises data for only 7 cancer types. The tool is also only capable of conducting survival analysis on limited number of datasets from the 7 cancer types. PrognoScan compiles data from 14 cancer types, but it does not contain data from TCGA, which is a very well organized and comprehensive repository of gene expression data. Also, Prognoscan cannot be used to study survival implications of multiple genes (signatures). In PROGgeneV2, we have attempted to provide a comprehensive survival analysis tool for research community to be able to perform biomarker identification with the largest repository of public datasets available in comparison to any other tool and several functionalities which were identified as being important from user feedback. We believe with the rich database and new functionalities which are not available in any other contemporary tool, PROGgeneV2 will prove to be a useful tool for scientific community.

## Availability and requirements

PROGgeneV2 is a web based tool available at http://www.compbio.iupui.edu/proggene. The tool is web based and platform independent.

## Electronic supplementary material

Additional file 1: Table S1: Datasets introduced in PROGgeneV2. For information about the datasets, please follow GSE ID's on GEO, or refer to specific publications on TCGA database. **Table S2.** Survival Variables and Covariates (if any) available for datasets added to the PROGgeneV2 database. **Figure S1.** KM plot created with PROGgeneV2 for WNT/CTNNB1 pathway in high risk ovarian cancer cohort (GSE32062). (DOCX 125 KB)

## Abbreviations

KM: Kaplan Meier
TCGA: The Cancer Genome Atlas
GEO: Gene Expression Omnibus
HR: Hazard Ratio
PROGgeneV1: PROGgene Version 1
PROGgeneV2: PROGgene Version 2.
