# Supplementary material for: PROGgeneV2: enhancements on the existing database
Source: BMC Cancer. 2014 Dec 17;14:970. doi: 10.1186/1471-2407-14-970 (PMC4300843; doi:10.1186/1471-2407-14-970)
Supplement: Supplementary file 1 — Additional file 1: Table S1: Datasets introduced in PROGgeneV2. For information about the datasets, please follow GSE ID's on GEO, or refer to specific publications on TCGA database. Table S2. Survival Variables and Covariates (if any) available for datasets added to the PROGgeneV2 database. Figure S1. KM plot created with PROGgeneV2 for WNT/CTNNB1 pathway in high risk ovarian cancer cohort (GSE32062). (DOCX 125 KB) [file 12885_2014_5146_MOESM1_ESM.docx]

Supplementary information for manuscript entitled " PROGgeneV2: Enhancements on the existing database."

Chirayu Pankaj Goswami and Harikrishna Nakshatri

Additional file 1: Table S1: Datasets introduced in PROGgeneV2. For information about the datasets, please follow GSE ID's on GEO, or refer to specific publications on TCGA database.

| **TISSUE** | **DATASET** | **DATASET DESCRIPTION** | **# SAMPLES** | **# GENES** |
| --- | --- | --- | --- | --- |
| ADRENAL | GSE19776 | GSE19776 - Adrenocortical Carcinoma Gene Expression Profiling | 22 | 21933 |
| BLADDER | GSE48276 | GSE48276 - Gene expression profiling of urothelial carcinoma | 73 | 20717 |
| BRAIN | GSE4412_U133A | freij-affy-human-91666 | 83 | 13720 |
| BRAIN | GSE4412_U133B | freij-affy-human-91666 | 83 | 10688 |
| BRAIN | GSE4271_U133B | Molecular subclasses of high-grade glioma: prognosis, disease progression, and neurogenesis | 78 | 10688 |
| BRAIN | GSE4271_U133A | Molecular subclasses of high-grade glioma: prognosis, disease progression, and neurogenesis | 77 | 13720 |
| BRAIN | GSE37418 | GSE37418 - Novel mutations target distinct subgroups of medulloblastoma. | 75 | 21703 |
| BRAIN | GSE16581 | GSE16581 - Genomic landscape of meningiomas: gene expression | 67 | 21703 |
| BRAIN | GSE42669 | GSE42669 - Patient specific orthotopic glioblastoma xenograft models recapitulate the histopathology and biology of human glioblastomas in situ (gene expression) | 55 | 21092 |
| BRAIN | GSE30074 | GSE30074 - Expression data from 30 medulloblastomas | 30 | 21103 |
| BRAIN | GSE2817 | GSE2817 - Wavelet modelling of microarray data provides chromosomal pattern of expression which predicts survival in gliomas | 25 | 21703 |
| BREAST | GSE48408 | GSE48408 - Long non-coding RNA HOTAIR is an independent prognostic marker of metastasis in estrogen receptor positive primary breast cancer | 164 | 21793 |
| BREAST | GSE42568 | GSE42568 - Breast Cancer Gene Expression Analysis | 104 | 21703 |
| BREAST | GSE37751 | GSE37751 - Molecular Profiles of Human Breast Cancer and Their Association with Tumor Subtypes and Disease Prognosis (Affymetrix) | 60 | 21093 |
| CERVICAL | GSE44001 | GSE44001 - Genetic profiling to predict recurrence of early cervical cancer | 300 | 20669 |
| COLON | GSE39582 | GSE39582 - Gene expression Classification of Colon Cancer defines six molecular subtypes with distinct clinical, molecular and survival characteristics [Expression] | 566 | 21703 |
| COLON | GSE14333 | GSE14333 - Expression data from 290 primary colorectal cancers | 187 | 21703 |
| COLON | GSE41258 | GSE41258 - Expression data from colorectal cancer patients | 182 | 13720 |
| COLON | GSE24551 | GSE24551 - Exon level expression profiling of colorectal cancer tissue samples | 160 | 14984 |
| COLON | GSE28722 | GSE28722 - EMT is the dominant program in human colon cancer (Agilent) | 125 | 15218 |
| COLON | GSE30378 | GSE30378 - Gene level expression profiling of colorectal cancer tissue samples (test sample series) | 95 | 14984 |
| COLON | GSE29621 | GSE29621 - mRNA and microRNA profile in colon cancer [mRNA data] | 65 | 21703 |
| COLON | GSE12945 | GSE12945 - Expression data from colorectal cancers | 62 | 13720 |
| COLON | GSE31595 | GSE31595 - Gene Expression Profiles in Stage II and III Colon Cancer. Application of a 128-gene signature | 37 | 21703 |
| COLON | GSE16125 | GSE16125 - Integrative approach for prioritizing cancer genes in sporadic colon cancer | 32 | 14984 |
| ESOPHAGUS | GSE19417 | GSE19417 - Human esophageal adenocarcinomas | 70 | 17367 |
| EYE | GSE22138 | GSE22138 - Expression Data from Uveal Melanoma primary tumors. | 63 | 21703 |
| EYE | GSE39717 | GSE39717 - Gene expression analysis of uveal melanoma tumor tissue | 30 | 21454 |
| HEME | GSE2658 | GSE2658 - Gene Expression Profiles of Multiple Myeloma | 546 | 21703 |
| HEME | GSE10846 | GSE10846 - Prediction of survival in diffuse large B cell lymphoma treated with chemotherapy plus Rituximab | 414 | 21703 |
| HEME | GSE16131_U133A | Differences Between Follicular Lymphoma With and Without Translocation t(14;18) | 180 | 13719 |
| HEME | GSE16131_U133B | Differences Between Follicular Lymphoma With and Without Translocation t(14;18) | 180 | 10686 |
| HEME | GSE4475 | GSE4475 - A Biologic Definition of Burkitt's Lymphoma from Transcriptional and Genomic Profiling | 158 | 13719 |
| HEME | GSE22762_U133P2 | An eight-gene expression signature for the prediction of survival and time to treatment in chronic lymphocytic leukemia | 107 | 21703 |
| HEME | GSE23501 | GSE23501 - DNA methylation signatures define molecular subtypes of Diffuse Large B Cell Lymphoma | 69 | 21703 |
| HEME | GSE22762_U133A | An eight-gene expression signature for the prediction of survival and time to treatment in chronic lymphocytic leukemia | 44 | 13719 |
| HEME | GSE22762_U133B | An eight-gene expression signature for the prediction of survival and time to treatment in chronic lymphocytic leukemia | 44 | 10684 |
| HNC | E-MTAB-1328 | E-MTAB-1328 - Methylome, transcriptome and miRNome profiling by array and high throughput sequencing of 89 patients with head and neck squamous cell carcinoma | 60 | 21703 |
| HNC | GSE10300 | GSE10300 - head and neck squamous cell carcinoma samples | 43 | 21703 |
| LIVER | GSE17856 | GSE17856 - Gene expression in nontumoral liver tissue and recurrence-free survival in hepatitis C virus-positive HCC | 43 | 14293 |
| LUNG | GSE30219 | GSE30219 - Off-context gene expression in lung cancer identifies a group of metastatic-prone tumors | 282 | 21703 |
| LUNG | GSE41271 | GSE41271 - Expression profiling of 275 lung cancer specimens | 275 | 25428 |
| LUNG | GSE31210 | GSE31210 - Gene expression data for pathological stage I-II lung adenocarcinomas | 226 | 21703 |
| LUNG | GSE50081 | GSE50081 - Validation of a histology-independent prognostic gene signature for early stage, non-small cell lung cancer including stage IA patients | 181 | 21703 |
| LUNG | GSE42127 | GSE42127 - Expression data for non-small-cell lung cancer | 176 | 25428 |
| LUNG | GSE13213 | GSE13213 - Relapse-related molecular signature in lung adenocarcinomas identifies patients with dismal prognosis | 117 | 30469 |
| LUNG | GSE3141 | GSE3141 - Lung Cancer Dataset | 111 | 21703 |
| LUNG | GSE37745 | GSE37745 - Biomarker discovery in non-small cell lung cancer: integrating gene expression profiling, meta-analysis and tissue microarray validation | 96 | 21703 |
| LUNG | GSE19188 | GSE19188 - Expression data for early stage NSCLC | 82 | 21703 |
| LUNG | GSE17710 | GSE17710 - Human lung squamous cell carcinoma expression profiling | 56 | 17083 |
| OVARIAN | GSE49997 | GSE49997 - Validating the Impact of a Molecular Subtype in Epithelial Ovarian Cancer (EOC) on Progression Free and Overall Survival | 194 | 16726 |
| OVARIAN | GSE17260 | GSE17260 - Prediction of progression-free survival in patients with advanced-stage serous ovarian cancer | 110 | 19566 |
| OVARIAN | GSE30161 | GSE30161 - Genomic Multivariate Predictors of Response to Adjuvant Chemotherapy in Ovarian Carcinoma: Predicting Platinum Resistance | 58 | 21703 |
| OVARIAN | GSE31245 | GSE31245 - Unique gene expression profile based upon pathologic response in epithelial ovarian cancer | 55 | 9651 |
| OVARIAN | GSE18520 | GSE18520 - Whole-genome oligonucleotide expression analysis of papillary serous ovarian adenocarcinomas | 53 | 21703 |
| OVARIAN | GSE32063 | GSE32063 - Immune-activation as a therapeutic direction for patients with high-risk ovarian cancer based on gene expression signature (2) | 40 | 19566 |
| OVARIAN | GSE23554 | GSE23554 - Ovarian Cancer Dataset | 28 | 13719 |
| PANCREAS | TCGA | TCGA - TCGA PAAD | 61 | 20502 |
| PANCREAS | GSE28735 | GSE28735 - Microarray gene-expression profiles of 45 matching pairs of pancreatic tumor and adjacent non-tumor tissues from 45 patients with pancreatic ductal adenocarcinoma | 42 | 21096 |
| PROSTATE | GSE16560 | GSE16560 - Molecular Sampling of Prostate Cancer: a dilemma for predicting disease progression | 281 | 6100 |
| RENAL | GSE33371 | GSE33371 - Beta-catenin status effects in human adrenocortical carcinomas (33), adenomas (22), and normal adrenal cortex (10) | 23 | 21703 |
| SKIN | GSE53118 | GSE53118 - BRAF Mutation, NRAS Mutation, and the Absence of an Immune-Related Expressed Gene Profile Predict Poor Outcome in Patients with Stage III Melanoma | 79 | 17617 |
| SKIN | GSE22153 | GSE22153 - Gene Experssion Profiling-Based Identification of Molecular Subtypes in Stage IV Melanoma with Different Clinical Outcome (test set) | 57 | 24614 |
| SKIN | GSE19234 | GSE19234 - Immune profile and mitotic index of metastatic melanoma lesions enhance clinical staging in predicting patient survival. | 44 | 21703 |

Additional file 1: Table S2: Survival Variables and Covariates (if any) available for datasets added to the PROGgeneV2 database.

| **DATASET** | **TISSUE** | **SURVIVAL VARIABLES** | **COVARIATES** |
| --- | --- | --- | --- |
| GSE16581 | BRAIN | OVERALL | AGE, GENDER |
| GSE2817 | BRAIN | OVERALL | AGE, GENDER |
| GSE30074 | BRAIN | OVERALL | AGE, GENDER |
| GSE37418 | BRAIN | OVERALL | GENDER, STAGE |
| GSE42669 | BRAIN | OVERALL | AGE, GENDER |
| GSE4271_U133A | BRAIN | OVERALL | AGE, GENDER, GRADE |
| GSE4271_U133B | BRAIN | OVERALL | AGE, GENDER, GRADE |
| GSE4412_U133A | BRAIN | OVERALL | AGE, GENDER, GRADE |
| GSE4412_U133B | BRAIN | OVERALL | AGE, GENDER, GRADE |
| GSE12945 | COLON | OVERALL | AGE, GENDER, TNMSTAGE, GRADE, UICC_STAGE |
| GSE14333 | COLON | RELAPSE FREE | AGE, GENDER, STAGE |
| GSE16125 | COLON | OVERALL | AGE, GENDER, STAGE |
| GSE24551 | COLON | OVERALL | STAGE |
| GSE28722 | COLON | OVERALL , METASTASIS FREE | AGE, STAGE |
| GSE30378 | COLON | OVERALL | STAGE |
| GSE31595 | COLON | RELAPSE FREE | AGE, GENDER, STAGE, CHEMOTHERAPY |
| GSE41258 | COLON | OVERALL | AGE, GENDER, STAGE, TNM_STAGE |
| GSE19417 | ESOPHAGUS | OVERALL | GENDER |
| GSE22138 | EYE | METASTASIS FREE | AGE, GENDER |
| GSE39717 | EYE | METASTASIS FREE | AGE, GENDER |
| GSE10846 | HEME | OVERALL | AGE, GENDER, STAGE, CHEMOTHERAPY |
| GSE16131_U133A | HEME | OVERALL | STAGE |
| GSE16131_U133B | HEME | OVERALL | STAGE |
| GSE22762_U133A | HEME | OVERALL |  |
| GSE22762_U133B | HEME | OVERALL |  |
| GSE22762_U133P2 | HEME | OVERALL |  |
| GSE23501 | HEME | OVERALL , RELAPSE FREE | AGE, GENDER |
| GSE2658 | HEME | OVERALL |  |
| GSE4475 | HEME | OVERALL | AGE, GENDER, STAGE, CHEMOTHERAPY, RADIOTHERAPY |
| E-MTAB-1328 | HNC | METASTASIS FREE | AGE, GENDER, STAGE |
| GSE10300 | HNC | RELAPSE FREE |  |
| GSE17856 | LIVER | RELAPSE FREE |  |
| GSE13213 | LUNG | OVERALL | AGE, GENDER, STAGE, TNM_STAGE, EGFR_MUTATION, KRAS_MUTATION, P53_MUTATION |
| GSE17710 | LUNG | OVERALL , RELAPSE FREE | AGE, GENDER, STAGE, GRADE |
| GSE30219 | LUNG | OVERALL | AGE, GENDER |
| GSE31210 | LUNG | OVERALL , RELAPSE FREE | AGE, GENDER, STAGE |
| GSE3141 | LUNG | OVERALL |  |
| GSE37745 | LUNG | OVERALL , RELAPSE FREE | AGE, GENDER, STAGE, CHEMOTHERAPY |
| GSE42127 | LUNG | OVERALL | AGE, GENDER, STAGE, CHEMOTHERAPY |
| GSE19188 | LUNG | OVERALL | GENDER |
| GSE17260 | OVARIAN | OVERALL , RELAPSE FREE | STAGE, GRADE |
| GSE18520 | OVARIAN | OVERALL |  |
| GSE23554 | OVARIAN | OVERALL | GRADE |
| GSE30161 | OVARIAN | OVERALL , RELAPSE FREE | AGE, STAGE, GRADE |
| GSE31245 | OVARIAN | OVERALL |  |
| GSE32063 | OVARIAN | OVERALL , RELAPSE FREE | STAGE, GRADE |
| GSE28735 | PANCREAS | OVERALL |  |
| TCGA | PANCREAS | OVERALL | AGE, GENDER, STAGE, GRADE, RADIOTHERAPY |
| GSE16560 | PROSTATE | OVERALL |  |
| GSE33371 | RENAL | OVERALL | AGE, GENDER, STAGE |
| GSE19234 | SKIN | OVERALL | AGE, GENDER, STAGE |
| GSE22153 | SKIN | OVERALL | AGE, GENDER, STAGE |
| GSE19776 | ADRENAL | OVERALL |  |
| GSE48276 | BLADDER | OVERALL | AGE, GENDER |
| GSE37751 | BREAST | OVERALL | AGE, STAGE, GRADE, ER, TRIPLE_NEG, CHEMOTHERAPY, HORMONAL_THERAPY |
| GSE42568 | BREAST | OVERALL , RELAPSE FREE | AGE, GRADE, ER |
| GSE48408 | BREAST | METASTASIS FREE | AGE, GRADE, ER |
| GSE44001 | CERVICAL | RELAPSE FREE | STAGE |
| GSE29621 | COLON | OVERALL | GENDER, STAGE, GRADE |
| GSE39582 | COLON | RELAPSE FREE | AGE, GENDER, CHEMOTHERAPY, BRAF_MUTATION, KRAS_MUTATION, P53_MUTATION |
| GSE41271 | LUNG | OVERALL , RELAPSE FREE | GENDER, STAGE |
| GSE50081 | LUNG | OVERALL , RELAPSE FREE | AGE, GENDER, STAGE |
| GSE49997 | OVARIAN | OVERALL , METASTASIS FREE | AGE, GRADE |
| GSE53118 | SKIN | OVERALL | AGE, GENDER, STAGE |

Additional file 1: Figure S1: KM plot created with PROGgeneV2 for WNT/CTNNB1 pathway in high risk ovarian cancer cohort (GSE32062 [1])


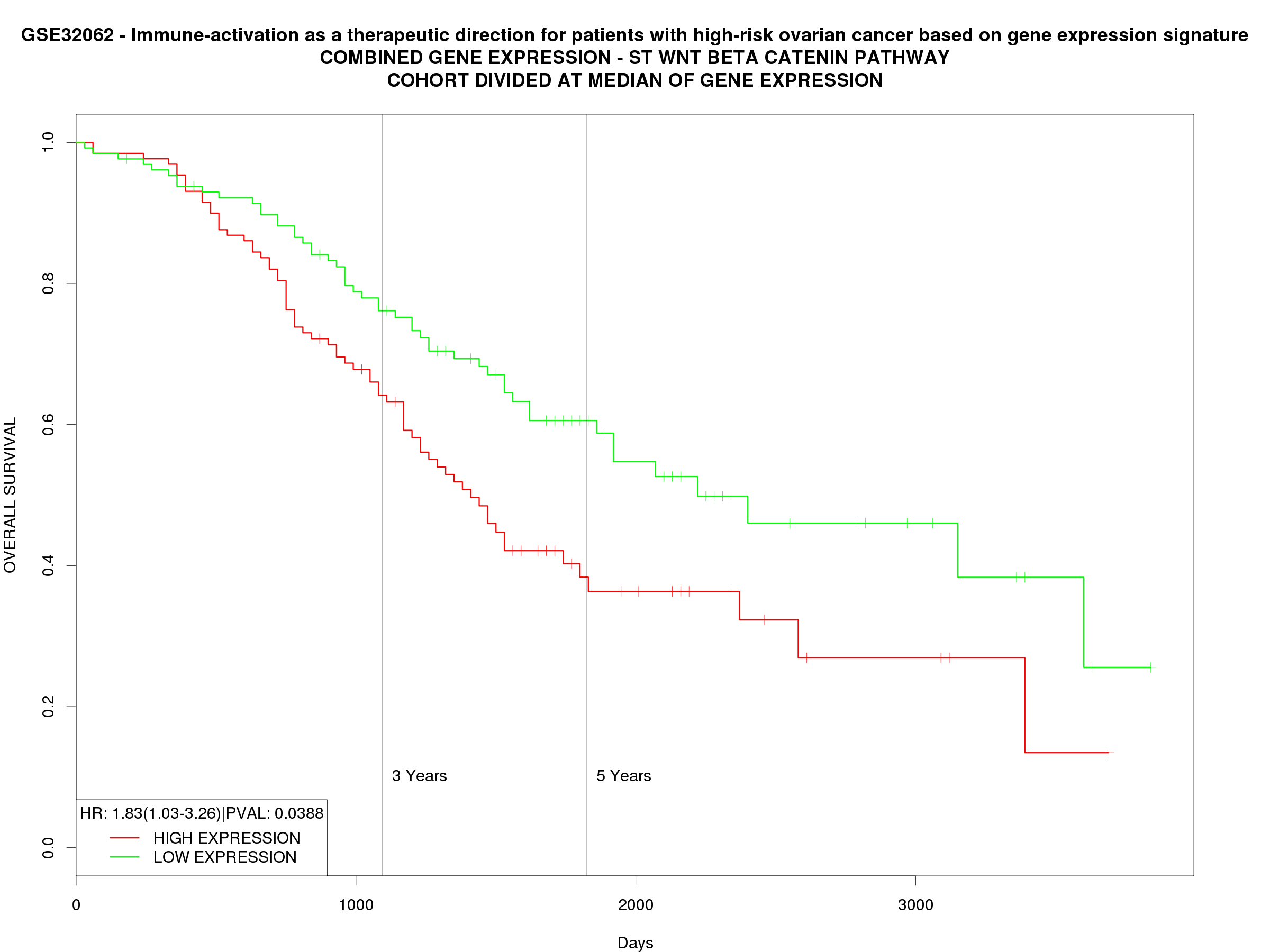


References

1. Yoshihara K, Tsunoda T, Shigemizu D, Fujiwara H et al. High-risk ovarian cancer based on 126-gene expression signature is uniquely characterized by downregulation of antigen presentation pathway. Clin Cancer Res 2012 Mar 1;18(5):1374-85.
